# Systematic Assessment of Risk of Fever in Solid Tumor Patients Treated With PD-1/PD-L1 Inhibitors: A Systematic Review and Meta-Analysis

**DOI:** 10.3389/fonc.2020.570080

**Published:** 2020-10-29

**Authors:** Hongmei Liu, Dongmei Xu, Wentao Wang, Fengchao Sun, Shuisheng Zhang, Xiaowei Yang, Yuan Tian

**Affiliations:** ^1^Department of Radiotherapy Oncology, Shandong Provincial Qianfoshan Hospital, The First Hospital Affiliated With Shandong First Medical University, Jinan, China; ^2^Nursing Department, Shandong Provincial Qianfoshan Hospital, The First Hospital Affiliated With Shandong First Medical University, Jinan, China; ^3^Department of Liver Transplantation and Hepatic Surgery, Shandong Provincial Qianfoshan Hospital, The First Hospital Affiliated With Shandong First Medical University, Jinan, China; ^4^Department of Pediatrics, The People's Hospital of Yuncheng County, Heze, China; ^5^Department of General Surgery, Peking University Third Hospital, Beijing, China; ^6^Department of Hepatobiliary Intervention, Beijing Tsinghua Changgung Hospital, School of Clinical Medicine, Tsinghua University, Beijing, China

**Keywords:** fever, PD-1, PD-L1, solid tumor, meta-analysis

## Abstract

**Purpose:** Drug-induced fever is frequently reported in cancer patients treated with anti-programmed cell death 1 (PD-1)/programmed cell death ligand 1 (PD-L1), and stoppage of the offending agent is the management of choice. However, given the complex management of cancer patients, this needs to be carefully studied. Therefore, we conducted a meta-analysis to estimate the risk of fever associated with anti-PD-1/PD-L1 in cancer patients.

**Methods:** From May 2010 to 2020, an electronic search was conducted through PubMed for relevant studies. All clinical trials reporting fever in cancer patients treated with PD-1/PD-L1 inhibitors were included, while other designs were excluded. A manual search was also conducted to search for relevant articles. Outcomes included the risk of pyrexia and febrile neutropenia in the overall population and based on the grade of fever (all grades vs. grades 3–5). The Newcastle–Ottawa Scale was used to assess the quality of included studies.

**Results:** Thirty-one articles, involving 27 clinical trials and 15,867 participants, were included. The increased risk of pyrexia for all grades is only found when PD-1/PD-L1 plus cytotoxic T lymphocyte-associated protein 4 (CTLA-4) was compared to CTLA-4 [odds ratio (OR) = 2.48, 95% CI: 1.17, 5.23]. The risk of febrile neutropenia for all-grade fever was significantly lower in the PD-1/PD-L1 group compared to that of chemotherapy alone (OR = 0.02, 95% CI: 0.01, 0.05). A similar trend in the risk of febrile neutropenia was also found for grades 3–5 (OR = 0.02, 95% CI: 0.01, 0.05).

**Conclusion:** The increased risk of pyrexia for all grades could only be found when PD-1/PD-L1 plus CTLA-4 was compared with CTLA-4. Meanwhile, compared to chemotherapy, PD-1/PD-L1 inhibitors reduced the risk of febrile neutropenia.

## Introduction

Fever, relating to drug, infections, or others, is frequently reported among cancer patients (1–3). In the course of antitumor therapy, drug-induced fever might be caused by chemotherapy (4), targeted therapy (5), or immunotherapy drugs (6). At present, in clinical practice, enough attention has been paid to drug-induced fever of cancer patients, and relevant guidelines have been developed in this regard (1–3). In order to control the fever of cancer patients in a timely manner, it is important to clarify the origin of the fever (1–6). In terms of drug-induced fever, stopping the offending agent remains the first choice of treatment (1–3). However, due to the delicate medical care in cancer patients, the sudden stoppage of antitumor therapy is very likely to lead to the rapid progression of such tumors. Therefore, the decision to stop antitumor drug-induced fever needs to be studied thoroughly.

In many clinical trials, immunotherapeutic drugs, especially those directed at the programmed cell death 1 (PD-1) and programmed cell death ligand 1 (PD-L1) pathways (7–37), have achieved satisfactory clinical efficacy and safety profiles in cancer patients. That being said, various treatment-emergent adverse events (TEAEs) were gradually discovered and reported in individual studies, including fever (7–37). However, to date, there has been no published systematic literature review studying the incidence or risk of drug-induced fever in cancer patients treated with PD-1/PD-L1 inhibitors (7–37). Therefore, in order to provide clear evidence in this regard, we conducted the current systematic review and meta-analysis to report the overall risk of drug-induced fever in cancer patients treated with PD-1/PD-L1 inhibitors.

## Methods

This systematic review and meta-analysis was carried out in accordance with the Preferred Reporting Items for Systematic Reviews and Meta-Analyses (PRISMA) guidelines (38).

### Types of Enrolled Studies

Clinical trials involving hematological malignancies were excluded first. Phase III clinical trials reporting the incidence or the risk of fever in patients with PD-1/PD-L1-positive solid tumors were prioritized. Clinical trials of other phases would be placed in an alternative position. All clinical trials had to have a control group to be eligible for inclusion in our study. At least one piece of fever-related data had to be available for inclusion; otherwise, the paper would be excluded. Various definitions of fever, such as pyrexia and febrile illness, were used to search for eligible studies, and any study reporting any definition of fever while being consistent with our eligibility criteria was included. We included articles that were originally published in the English language, while other trials published in other languages were excluded.

### Search Strategy

A systematic electronic search was carried out for relevant clinical trials (reporting fever in cancer patients treated with anti-PD-1/PD-L1 agents) that were published in the past 10 years (May 29, 2010, to May 29, 2020) through PubMed using a set of keywords, as follows: “neoplasm,” “cancer,” “precancer,” “pre-cancer,” “malignant,” “premalignant,” “tumor,” “PD1/PD-L1,” “nivolumab,” “Opdivo,” “pembrolizumab,” “Keytruda,” “Imfinzi,” “MK-3475,” “atezolizumab,” “Tecentriq,” “MPDL3280A,” “avelumab,” “Bavencio,” “durvalumab,” “camrelizumab,” and “BMS-963558” (39). Three independent reviews carried out the electronic search phase of relevant human-limited literature that was published in English according to the aforementioned criteria. Eligibility and duplicates were checked independently by three reviewers (Dongmei Xu, Hongmei Liu, and Wentao Wang). Any discrepancies were solved through discussion among reviewers, and when needed, a senior reviewer would give the final decision on the matter. The baseline characteristics of all included clinical trials are summarized and presented in Table 1.

**Table 1:** Baseline characteristics of the included clinical trials (*n* = 27).

**No**.	**Reference**	**NCT number**	**Drug name**	**Treatment regimen**	**Involving patients**	**Pyrexia**	**Febrile neutropenia**	**Previous therapy**	**Phase**	**RCT**	**Tumor type**
1	Huang et al. (7)	NCT03099382 (ESCORT)	Camrelizumab (PD-1)	Camrelizumab vs. (docetaxel or irinotecan)	448	N/A	5	YES	III	YES	Oesophageal squamous cell carcinoma (OSCC)
2	Motzer et al. (8)	NCT02684006 (JAVELIN)	Avelumab (PD-L1)	Avelumab plus axitinib vs. sunitinib	873	118	N/A	NO	III	YES	Advanced renal-cell carcinoma
3	Ascierto et al. (9)	NCT02130466	Pembrolizumab (PD-1)	Pembrolizumab + dabrafenib + trametinib (DT) vs. dabrafenib + trametinib (DT)	120	91	N/A	NO	II	YES	Melanoma
4	Cohen et al. (10)	NCT02252042 (KEYNOTE-040)	Pembrolizumab (PD-1)	Pembrolizumab vs. (methotrexate, docetaxel, cetuximab)	480	9	N/A	YES	III	YES	Head and neck squamous cell carcinoma (HNSCC)
5	Kato et al. (11)	NCT02569242 (ATTRACTION-3)	Nivolumab (PD-1)	Nivolumab vs. (paclitaxel or docetaxel)	417	N/A	22	YES	III	YES	Oesophageal squamous cell carcinoma (OSCC)
6	Burtness et al. (12)	NCT02358031 (KEYNOTE-048)	Pembrolizumab (PD-1)	Pembrolizumab alone or with chemotherapy (a platinum and 5-fluorouracil) vs. cetuximab + chemotherapy (a platinum and 5-fluorouracil)	863	154	N/A	NO	III	YES	Head and neck squamous cell carcinoma (HNSCC)
7	Paz-Ares et al. (13)	NCT03043872 (CASPIAN)	Durvalumab (PD-L1)	Durvalumab + platinum–etoposide (EP) vs. platinum-etoposide (EP)	531	N/A	34	NO	III	YES	SCLC
8	Schmid et al. (14)	NCT02425891 (IMpassion130)	Atezolizumab (PD-L1)	Atezolizumab + nab-paclitaxel vs. placeb + Nab-paclitaxel	890	132	N/A	NO	III	YES	Breast cancer
9	Horn et al. (15)	NCT02763579 (IMpower133)	Atezolizumab (PD-L1)	Atezolizumab + carboplatin and etoposide (EC) vs. placebo +carboplatin and etoposide (EC)	394	N/A	18	NO	III	YES	SCLC
10	Socinski et al. (16)	NCT02366143 (IMpower150)	Atezolizumab (PD-L1)	Atezolizumab + bevacizumab plus carboplatin plus paclitaxel (BCP) vs. bevacizumab plus carboplatin plus paclitaxel (BCP)	787	N/A	61	NO	III	YES	NSCLC
11	Paz-Ares et al. (17)	NCT02775435 (KEYNOTE-407)	Pembrolizumab (PD-1)	Pembrolizumab + carboplatin and either paclitaxel or nanoparticle albumin-bound [nab]–paclitaxel (CP) vs. carboplatin and either paclitaxel or nanoparticle albumin-bound [nab]–paclitaxel (CP)	558	85	N/A	NO	III	YES	NSCLC
12	Barlesi et al. (18)	NCT02395172 (JAVELIN Lung 200)	Avelumab (PD-L1)	Avelumab vs. docetaxel	792	48	37	YES	III	YES	NSCLC
13	Hida et al. (19)	NCT02008227	Atezolizumab (PD-L1)	Atezolizumab vs. docetaxel	101	26	16	YES	III	YES	NSCLC
14	Gandhi et al. (20)	NCT02578680 (KEYNOTE-189)	Pembrolizumab (PD-1)	Pembrolizumab + pemetrexed and a platinum- based drug vs. pemetrexed and a platinum-based drug	439	109	N/A	NO	III	YES	NSCLC
15	Antonia et al. (21)	NCT02125461	Durvalumab (PD-L1)	Durvalumab vs. placebo	709	94	N/A	YES	III	YES	NSCLC
16	Kang et al. (22)	NCT02267343 (ATTRACTION-2)	Nivolumab (PD-1)	Nivolumab vs. placebo	491	43	N/A	YES	III	YES	Gastric or junction cancer
17	Bellmunt et al. (23)	NCT02256436 (KEYNOTE-045)	Pembrolizumab (PD-1)	Pembrolizumab vs. platinum-based chemotherapy (paclitaxel, docetaxel, or vinflunine)	521	69	19	YES	III	YES	Urothelial carcinoma (UC)
18	Rittmeyer et al. (24)	NCT02008227 (OAK)	Atezolizumab (PD-L1)	Atezolizumab vs. docetaxel	1187	184	62	YES	III	YES	NSCLC
19	Langer et al. (25)	NCT02039674 (KEYNOTE-021)	Pembrolizumab (PD-1)	Pembrolizumab + pembrolizumab + carboplatin vs. pembrolizumab + carboplatin	121	N/A	1	NO	II	YES	NSCLC
20	Reck et al. (26)	NCT02142738 (KEYNOTE-024)	Pembrolizumab (PD-1)	Pembrolizumab vs. platinum-based chemotherapy	304	24	N/A	NO	III	YES	NSCLC
21	Ferris et al. (27)	NCT02105636 (CheckMate 141)	Nivolumab (PD-1)	Nivolumab vs. (methotrexate, docetaxel, or cetuximab)	347	7	N/A	YES	III	YES	Head and neck squamous cell carcinoma (HNSCC)
22	Herbst et al. (28)	NCT01905657 (KEYNOTE-010)	Pembrolizumab (PD-1)	Pembrolizumab vs. docetaxel	991	41	15	YES	II/III	YES	NSCLC
23	Hodi et al. (29)	NCT01927419 (CheckMate 069)	Nivolumab (PD-1)	Nivolumab + ipilimumab vs. ipilimumab	140	23	1	NO	III	YES	Melanoma
24	Borghaei et al. (30)	NCT01673867 (CheckMate 057)	Nivolumab (PD-1)	Nivolumab vs. docetaxel	555	25	27	YES	III	YES	NSCLC
25	Brahmer et al. (31)	NCT01642004 (CheckMate 017)	Nivolumab (PD-1)	Nivolumab vs. docetaxel	260	16	14	YES	III	YES	NSCLC
26	Antonia et al. (32)	NCT02125461 (PACIFIC)	Durvalumab (PD-L1)	Durvalumab vs. placebo	709	91	N/A	YES	III	YES	NSCLC
27	Powles et al. (33)	NCT02302807 (IMvigor211)	Atezolizumab (PD-L1)	Atezolizumab vs. chemotherapy (physician's choice: vinflunine, paclitaxel, or docetaxel)	902	65	26	YES	III	YES	Locally advanced or metastatic urothelialcarcinoma (UC)
28	Wolchok et al. (34)	NCT01844505 (CheckMate 067)	Nivolumab (PD-1)	Nivolumab vs. ipilimumab or nivolumab + ipilimumab	937	102	N/A	NO	III	YES	Melanoma
29	Hodi et al. (35)	NCT01844505 (CheckMate 067)	Nivolumab (PD-1)	Nivolumab plus ipilimumab or nivolumab alone vs. ipilimumab alone	937	102	1	NO	III	YES	Advanced melanoma
30	Larkin et al. (36)	NCT01844505 (CheckMate 067)	Nivolumab (PD-1)	Nivolumab plus ipilimumab or nivolumab alone vs. ipilimumab alone	937	97	N/A	NO	III	YES	Advanced melanoma
31	Motzer et al. (8)	NCT01844505 (CheckMate 067)	Nivolumab (PD-1)	Nivolumab plus ipilimumab or nivolumab alone vs. ipilimumab alone	937	102	N/A	NO	III	YES	Advanced melanoma

*RCT, randomized controlled trial; N/A, not available*.

### Evaluation of Study Quality and Publication Bias

Publication bias was assessed using Egger's regression test, while the quality of the included trials was assessed by the Newcastle–Ottawa Scale, which was proposed by the Cochrane Collaboration (38, 40–43). The quality assessment of included clinical trials was also carried out by the above three reviewers (Dongmei Xu, Hongmei Liu, and Wentao Wang). The quality assessment included assessing the risk of bias in the following points: random sequence generation, allocation concealment, blinding of participants and personnel, blinding of outcome assessment, incomplete outcome data, and selective outcome reporting. All these points were evaluated together, and the evaluation results would be summarized in a single graph. Harbord's test was used to check publication bias for all enrolled clinical trials (44) A *P* < 0.05 was considered indicative of publication bias.

### Exposure and Outcome of Interest

Basic information of enrolled studies, including the first author's name, year of publication, trial number, trial title, the specific name of the anti-PD-1/PD-L1 agent, status of previous therapies, treatment regimens, number of participants, and number of fever events (rate), was collected and summarized in Table 1. Various definitions and terms of fever indicators, such as pyrexia and febrile neutropenia, were collected and used for the final comprehensive analysis. Both all-grade and grade 3–5 fever data were used for the final meta-analysis.

### Assessment of Heterogeneity and Statistical Analysis

Cochrane's Q statistic and the *I*^2^ statistic were used to check the heterogeneity among analyzed studies, as proposed by Higgins et al. (38, 39, 43). The grade of heterogeneity was evaluated by the range of *I*^2^ values (38, 43). Heterogeneity was taken as low, moderate, or high according to *I*^2^ values <25, 25–50, and >50%, respectively (39). Odds ratio (OR) and the corresponding 95% confidence interval (CI) would be calculated by random effect (RE) (45). A *P* < 0.05 was considered the cutoff value for statistical significance. In order to clarify the relationship between fever indicators (pyrexia and febrile neutropenia) and PD-1/PD-L1 inhibitors, we performed a large number of subgroup analyses based on the type of tumor, the treatment regimen, and the specific administered drug. The software (Review Manager 5.3) was used for data consolidation and analysis. Statistical tests were all two-sided (39).

## Results

### Literature Search Results

**Figure 1:**
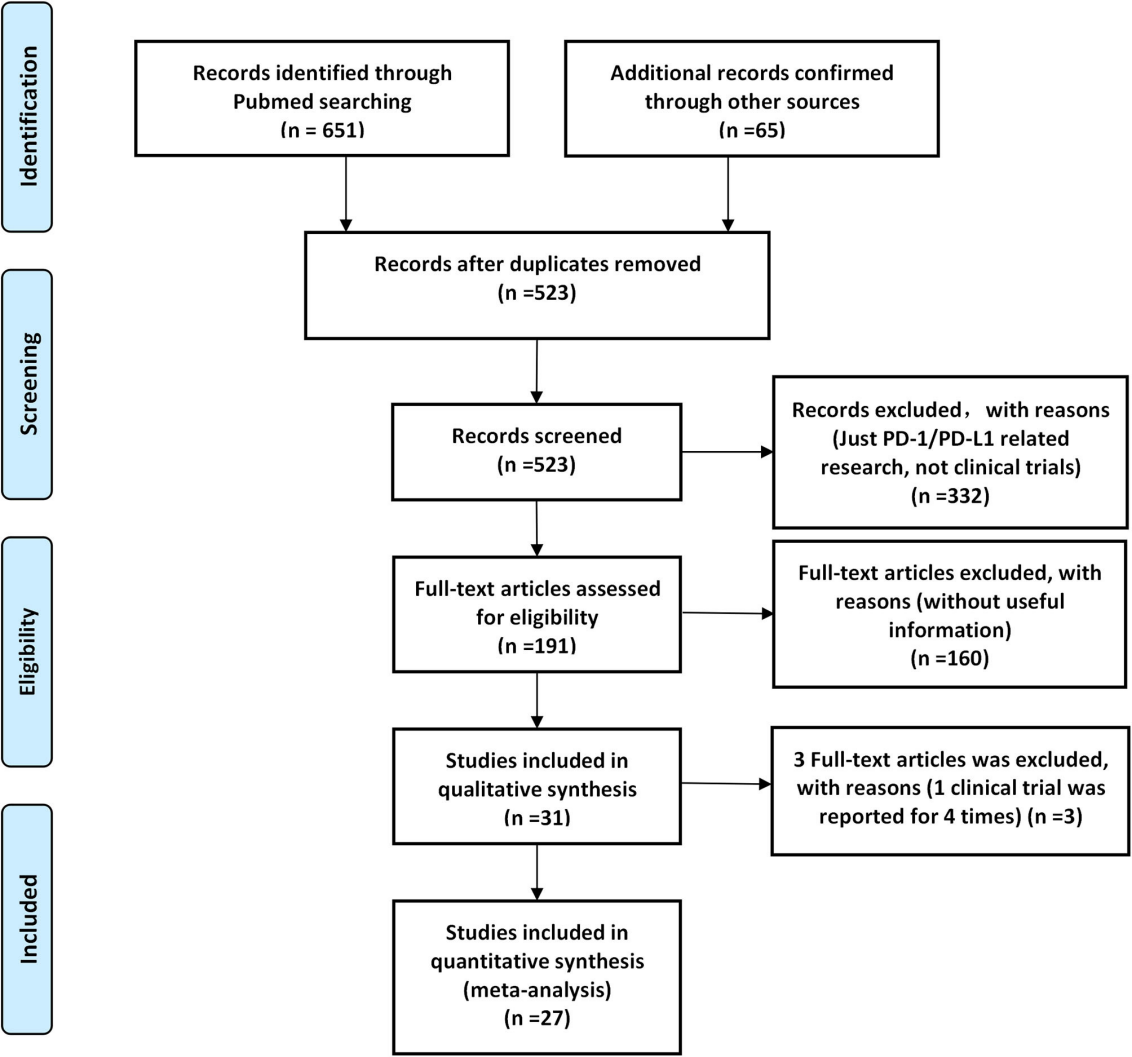
A Preferred Reporting Items for Systematic Reviews and Meta-Analyses (PRISMA) flow diagram of the screening process of the systematic review.

**Figure 2:**
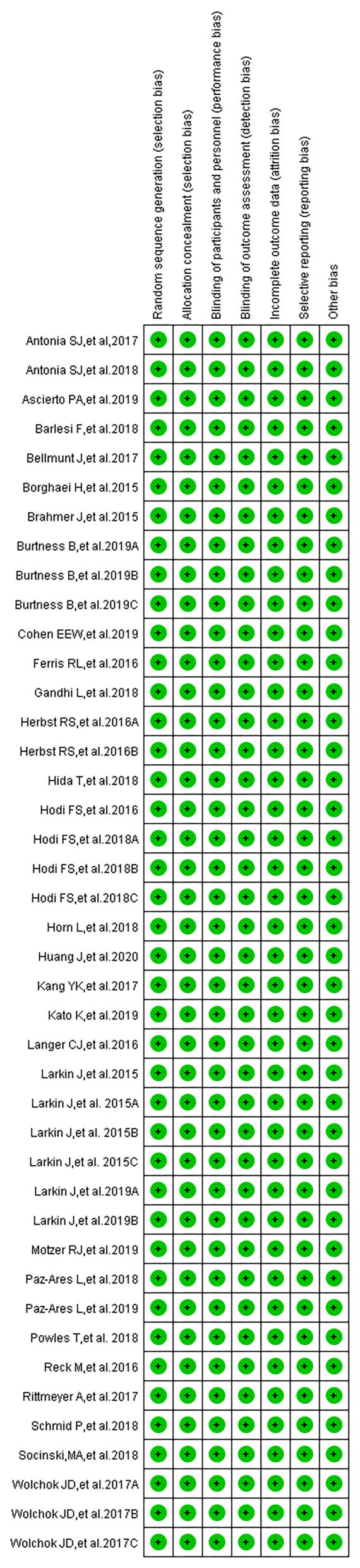
Risk of bias summary: review authors' judgments about each risk of bias item for each included study.

According to our preliminary electronic search, a total of 651 articles discussing PD-1/PD-L1 inhibitors and cancer, including clinical trials, were identified on PubMed, and 65 related studies were added after conducting a manual search of articles. A total of 31 published articles (27 clinical trials), including 15,867 participants, were finally included in our review. The basic information of included studies is listed and summarized in Table 1 (7–37). The PRISMA flow diagram of our review is shown in Figure 1, while the risk of bias summary is presented in Figure 2 (7–37). Different stages of a clinical trial, named CheckMate 067, were reported by four articles (8, 34–36). After reviewing and evaluating the data of the four articles, only the most comprehensive data provided by one of four articles were used for the final comprehensive analysis (35). After assessment and screening for all included clinical trials, the data relating to fever were mainly displayed in two forms: pyrexia and febrile neutropenia. The two variables would be analyzed separately.

### Characteristics of Identified Trials

Out of the 27 included clinical trials, 24 articles were phase III trials, two were phase II trials, and one article was a phase II/III trial (9, 25, 28). PD-1 inhibitors were used in 17 clinical trials (7, 9–12, 17, 20, 22, 23, 25–31, 35), while PD-L1 inhibitors were reported in the remaining 10 clinical trials (8, 13–16, 18, 19, 21, 24, 32, 33). The drugs that were used in each individual trial are listed as follows: camrelizumab (PD-1, *n* = 1) (7), nivolumab (PD-1, *n* = 7) (11, 22, 27, 29–31, 35), pembrolizumab (PD-1, *n* = 9) (9, 10, 12, 17, 20, 23, 25, 26, 28), avelumab (PD-L1, *n* = 2) (8, 12), durvalumab (PD-L1, *n* = 3) (13, 21, 32), and atezolizumab (PD-L1, *n* = 7) (14–16, 19, 24, 33). In terms of cancer type, 13 trials included patients with non-small-cell lung cancer (NSCLC) (16–21, 24–26, 28, 30–32), two trials included patients with small-cell lung cancer (SCLC) (13, 15), two trials included patients with esophageal squamous cell carcinoma (OSCC) (7, 11), three trials included patients with head and neck squamous cell carcinoma (HNSCC) (10, 12, 27), two trials included patients with urothelial cancer (UC) (23, 33), one trial included patients with breast cancer (BC) (14), three trials included patients with melanoma (9, 29, 35), one trial included patients with renal cell carcinoma (RCC) (8), and one trial included patients with gastric or junction cancer (22). PD-1/PD-L1 inhibitors were given as a first-line therapy in 13 clinical trials (8, 9, 12–17, 20, 25, 26, 29, 35), while platinum-based antitumor regimens were prescribed before PD-1/PD-L1 inhibitors in the remaining 14 trials (7, 10, 11, 18, 19, 21–24, 27, 28, 30–33).

### Risk of Bias

The overall risk of bias of all included studies is presented in a single graph (Figure 2, Supplementary Figure 2). Meanwhile, publication bias, evaluated by Harbord's test, shown in the form of funnel plots, was checked and displayed in Supplementary Figures 3–6 (38, 40–43).

### The Risk of Pyrexia

Among the included trials, pyrexia was reported in 21 trials (8–10, 12, 14, 17–24, 26–33, 35). The data were divided into five groups according to the treatment regimen: group A (PD-1/PD-L1 inhibitor + chemotherapy vs. chemotherapy) (14, 17, 20), group B (PD-1/PD-L1 inhibitor vs. chemotherapy alone) (10, 12, 18, 19, 23, 24, 26–28, 30, 31, 33), group C (PD-1/PD-L1 inhibitor vs. placebo) (21, 22, 32), group D (PD-1/PD-L1 inhibitor + targeted therapy vs. targeted therapy alone) (8, 9), and group E [PD-1 inhibitor vs. PD-1 inhibitor + cytotoxic T lymphocyte-associated protein 4 (CTLA-4)] (29, 35). Then, the risk of pyrexia for all grades and grades 3–5 was analyzed.

**Figure 3:**
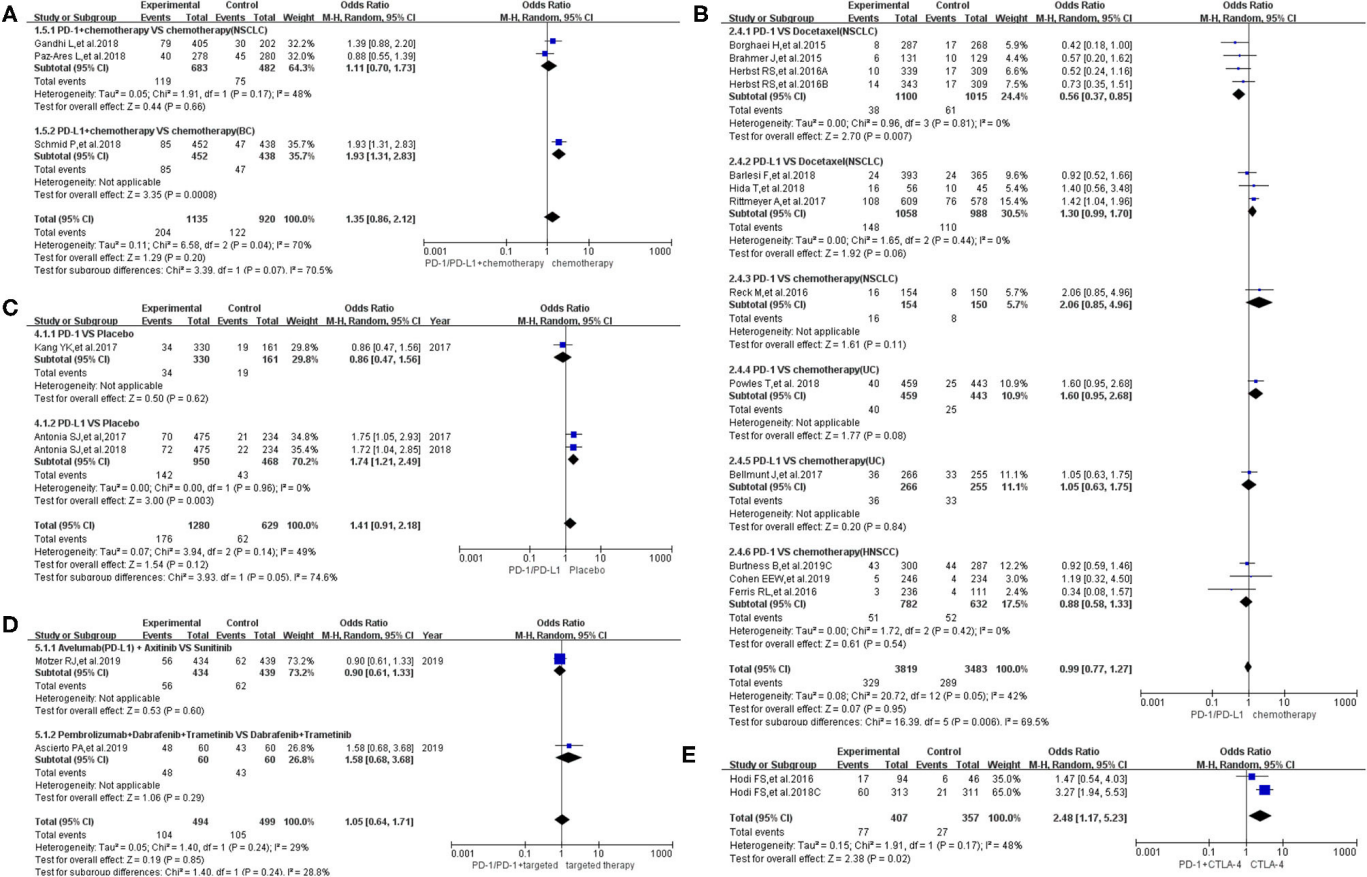
Forest plots of all-grade pyrexia. **(A)** The risk of all-grade pyrexia evaluated by random effect model [programmed cell death 1 (PD-1)/programmed cell death ligand 1 (PD-L1) + chemotherapy vs. chemotherapy]: subgroup analysis was put into practice based on tumor types in both groups. **(B)** The risk of all-grade pyrexia evaluated by random effect model (PD-1/PD-L1 vs. chemotherapy): subgroup analysis was put into practice based on the treatment regimen in both groups. **(C)** The risk of all-grade pyrexia evaluated by random effect model (PD-1/PD-L1 vs. placebo): subgroup analysis was put into practice based on the drug type of the experimental group. **(D)** The risk of all-grade pyrexia evaluated by random effect model (PD-1/PD-L1 + targeted therapy vs. targeted therapy): subgroup analysis was put into practice based on the involved drugs in both groups. **(E)** The risk of all-grade pyrexia evaluated by random effect model [PD-1/PD-L1 + cytotoxic T lymphocyte-associated protein 4 (CTLA-4) vs. CTLA-4].

Compared to chemotherapy (group A), our analysis revealed no statistically significant difference regarding the risk of pyrexia for all grades in cancer patients treated with PD-1/PD-L1 inhibitor plus chemotherapy (OR = 1.35, 95% CI: 0.86, 2.12; Figure 3A) (14, 17, 20). The analysis revealed high heterogeneity (*I*^2^ = 70%), which was suggested, by the subgroup analysis, to originate from the two included clinical trials on NSCLC (*I*^2^ = 48%; Figure 3A) (17, 20). The evaluation result of bias was shown in the form of funnel plots, which are present in Supplementary Figure 3A (14, 17, 20).

Similar to the above finding, there was no statistically significant difference regarding the risk of all-grade pyrexia in group B (OR = 0.99, 95% CI: 0.77, 1.27; Figure 3B), with moderate heterogeneity (*I*^2^ = 42%) (10, 12, 18, 19, 23, 24, 26–28, 30, 31, 33). That being said, a statistically significant difference in pyrexia risk was found in this particular subgroup (PD-1 vs. docetaxel) (OR = 0.56, 95% CI: 0.37, 0.85; Figure 3B) (28, 30, 31). In other words, the risk of pyrexia was obviously lower than that of the control group of docetaxel among NSCLC patients (28, 30, 31). Based on the tumor type, further subgroup analysis results were provided in Supplementary Figure 1. Through different subgrouping methods, we inferred that the moderate heterogeneity of the analysis results might come from the data themselves. The funnel plot is displayed in Supplementary Figure 3B.

Compared to placebo (group C), the risk of pyrexia was significantly higher in patients treated with PD-L1 inhibitor, in particular (OR = 1.74, 95% CI: 1.21, 2.49; Figure 3C), while no obvious statistically significant difference was noted for the overall group (PD-1/PD-L1 inhibitors) (OR = 1.41, 95% CI: 0.91, 2.18; Figure 3C), with moderate heterogeneity (*I*^2^ = 49%) (21, 22, 32). The results of the subgroup analysis suggested that the moderate heterogeneity might mainly originate from the included PD-1 clinical trial on gastric or junction cancer (Figure 3C) (22). The risk of bias is shown in the form of funnel plots in Supplementary Figure 3C (21, 22, 32).

The overall analysis results of group D revealed no statistically significant difference regarding pyrexia of all grades among the studied arms (OR = 1.05, 95% CI: 0.64, 1.71; Figure 3D) (8, 9). The funnel plot is provided in Supplementary Figure 3D.

Inconsistent with the above findings, the overall risk of pyrexia for all-grade fever in patients in group E was significantly different in the studied arms (OR = 2.48, 95% CI: 1.17, 5.23; Figure 3E) (29, 35). Moderate heterogeneity (*I*^2^ = 48%) was found among the included clinical trials. The corresponding funnel plot is provided in Supplementary Figure 3E (29, 35).

**Figure 4:**
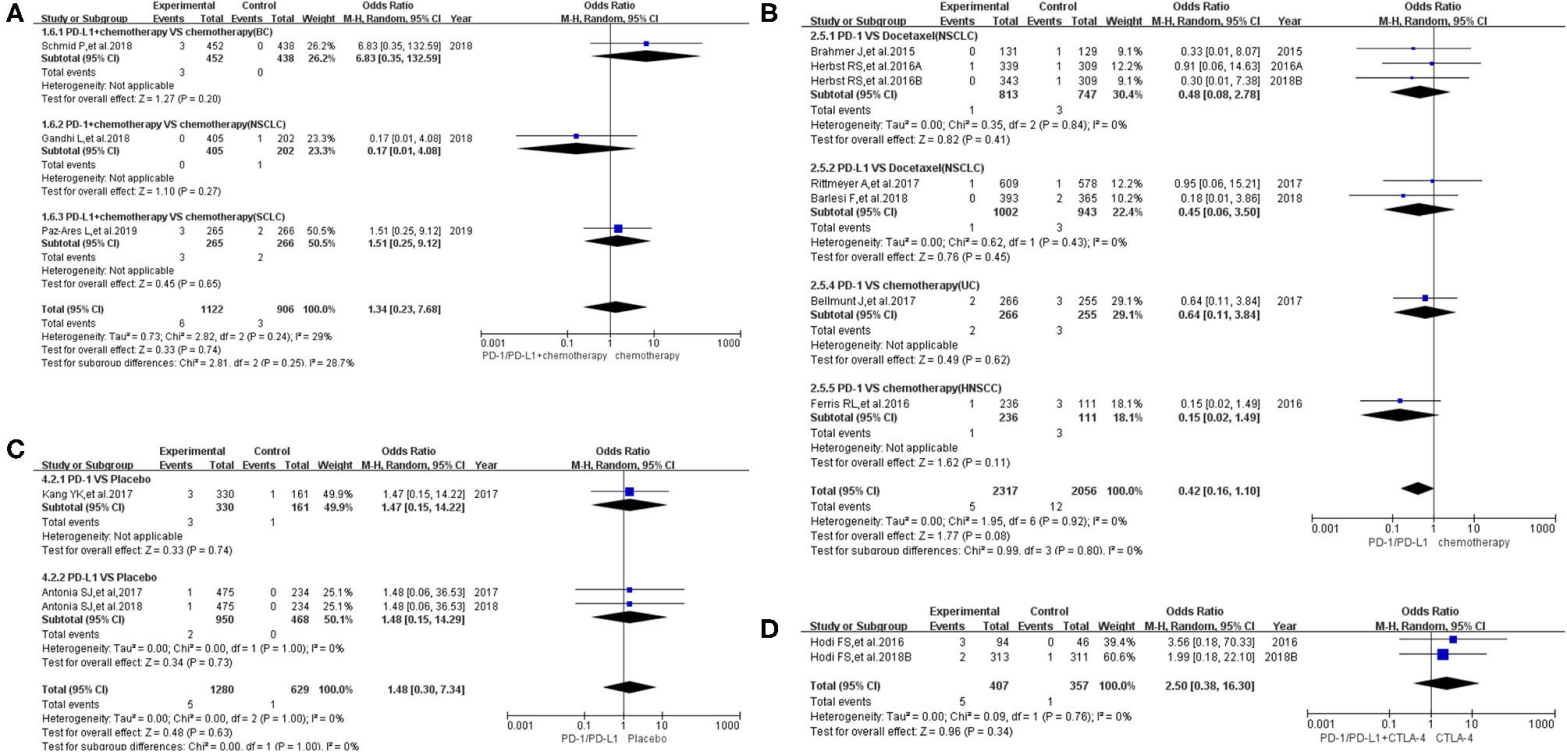
Forest plots of pyrexia for grades 3–5. **(A)** The risk of pyrexia for grade 3–5 fever evaluated by random effect model [programmed cell death 1 (PD-1)/programmed cell death ligand 1 (PD-L1) + chemotherapy vs. chemotherapy]: subgroup analysis was put into practice based on tumor types in both groups. **(B)** The risk of pyrexia for grade 3–5 fever evaluated by random effect model (PD-1/PD-L1 vs. chemotherapy): subgroup analysis was put into practice based on the treatment regimen and tumor type of the control group. **(C)** The risk of pyrexia for grade 3–5 fever evaluated by random effect model (PD-1/PD-L1 vs. placebo): subgroup analysis was put into practice based on the drug type of the experimental group. **(D)** The risk of pyrexia for grade 3–5 fever evaluated by random effect model [PD-1/PD-L1 + cytotoxic T lymphocyte-associated protein 4 (CTLA-4) vs. CTLA-4].

When the same grouping and analysis method were used to analyze the risk of pyrexia for grades 3–5, no statistically significant differences regarding the risk of pyrexia were found in each individual group (both overall and in different subgroups) (Figure 4) (8, 9, 13, 14, 18, 20–24, 26–29, 31, 32, 35). The corresponding funnel plots are provided in Supplementary Figure 4 (8, 9, 13, 14, 18, 20–24, 26–29, 31, 32, 35).

### The Risk of Febrile Neutropenia

Sixteen clinical trials reported data of febrile neutropenia and were included in the final comprehensive meta-analysis (7, 11, 13, 15, 16, 18, 19, 23–25, 28–31, 33, 35). The data were divided into three groups according to the treatment regimen: group A (PD-1/PD-L1 inhibitor + chemotherapy vs. chemotherapy) (13, 15, 16, 25), group B (PD-1/PD-L1 inhibitor vs. chemotherapy) (7, 11, 18, 19, 23, 24, 28, 30, 31, 33), and group C (PD-1/PD-L1 inhibitor + CTLA-4 vs. CTLA-4) (29, 35). Then, the risk of febrile neutropenia for all grades and grades 3–5 was checked.

**Figure 5:**
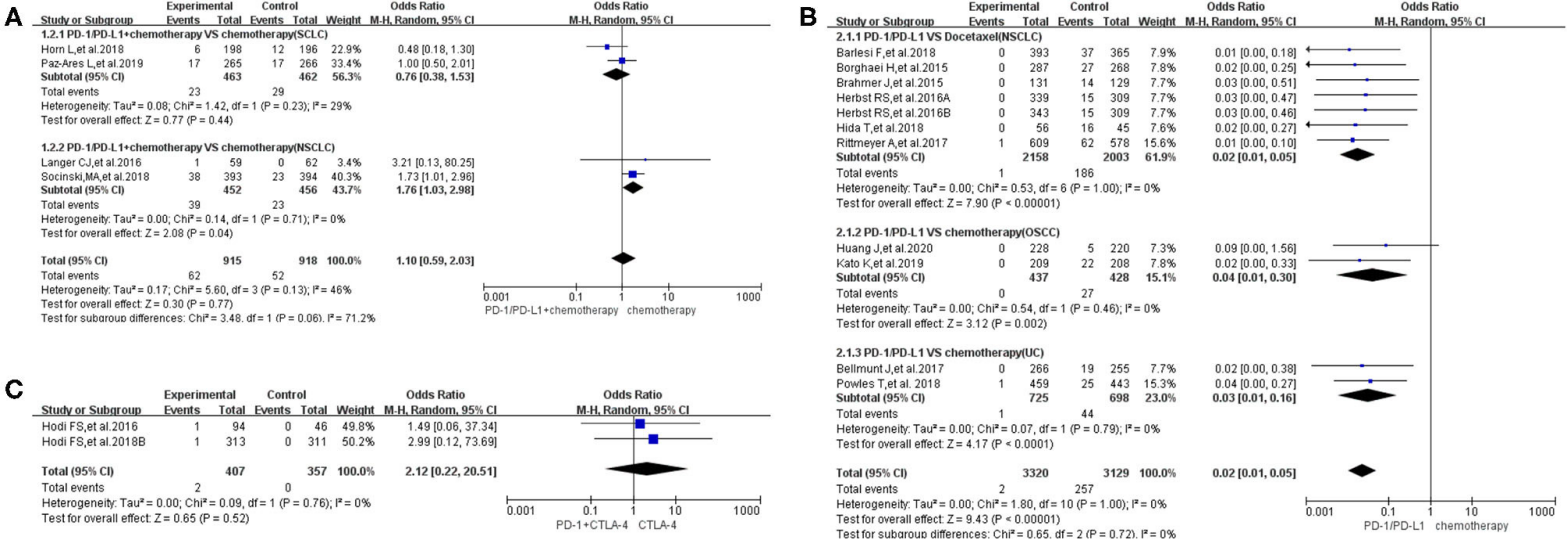
Forest plots of all-grade febrile neutropenia. **(A)** The risk of all-grade febrile neutropenia evaluated by random effect model [programmed cell death 1 (PD-1)/programmed cell death ligand 1 (PD-L1) + chemotherapy vs. chemotherapy]: subgroup analysis was put into practice based on tumor types in both groups. **(B)** The risk of all-grade febrile neutropenia evaluated by random effect model (PD-1/PD-L1 vs. chemotherapy): subgroup analysis was put into practice based on the treatment regimen and tumor type of the control group. **(C)** The risk of all-grade febrile neutropenia evaluated by random effect model [PD-1/PD-L1 + cytotoxic T lymphocyte-associated protein 4 (CTLA-4) vs. CTLA-4].

**Figure 6:**
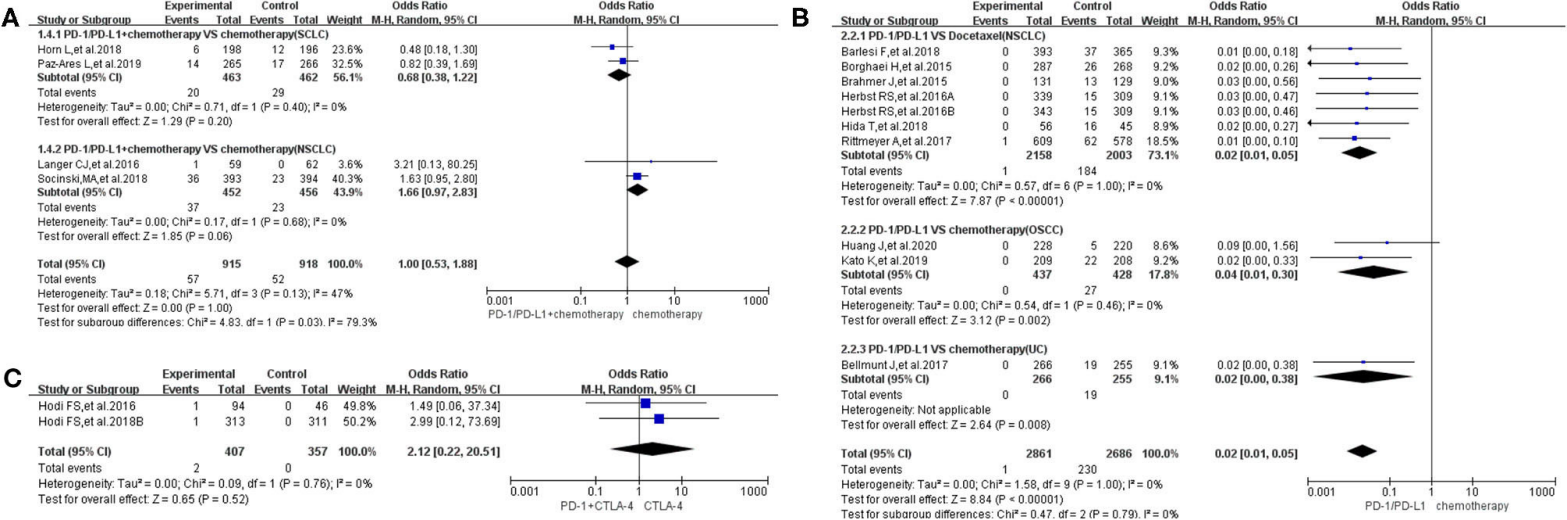
Forest plots of febrile neutropenia for grades 3–5. **(A)** The risk of febrile neutropenia for grade 3–5 fever evaluated by random effect model [programmed cell death 1 (PD-1)/programmed cell death ligand 1 (PD-L1) + chemotherapy vs. chemotherapy]: subgroup analysis was put into practice based on tumor types in both groups. **(B)** The risk of febrile neutropenia for grade 3–5 fever evaluated by random effect model (PD-1/PD-L1 vs. chemotherapy): subgroup analysis was put into practice based on the treatment regimen and tumor type of the control group. **(C)** The risk of febrile neutropenia for grade 3–5 fever evaluated by random effect model [PD-1/PD-L1 + cytotoxic T lymphocyte-associated protein 4 (CTLA-4) vs. CTLA-4].

In group A, our analysis revealed no statistically significant difference between chemotherapy alone and PD-1/PD-L1 plus chemotherapy regarding febrile neutropenia for all grades (OR = 1.10, 95% CI: 0.59, 2.03; Figure 5A), with moderate heterogeneity (*I*^2^ = 46%) (13, 15, 16, 25). The results of the subgroup analysis suggested that the encountered moderate heterogeneity might mainly originate from the two included clinical trials on SCLC (*I*^2^ = 29%; Figure 5A) (13, 15). The evaluation of the risk of bias is shown in the form of funnel plots in Supplementary Figure 5A (13, 15, 16, 25). A similar trend in the risk of febrile neutropenia could also be seen when the data of grades 3–5 alone were analyzed (OR = 1.00, 95% CI: 0.53, 1.88; Figure 6A) (13, 15, 16, 25). Moderate heterogeneity (*I*^2^ = 47%) was found. The corresponding funnel plot is displayed in Figure 6A (13, 15, 16, 25).

In group B, the risk of febrile neutropenia for all grades was significantly lower in the PD-1/PD-L1 inhibitor group compared to that of the control group (chemotherapy) (OR = 0.02, 95% CI: 0.01, 0.05; Figure 5B) (7, 11, 18, 19, 23, 24, 28, 30, 31, 33). A similar trend in the risk of febrile neutropenia could also be found for grades 3–5 (OR = 0.02, 95% CI: 0.01, 0.05; Figure 6B) (7, 11, 18, 19, 23, 24, 28, 30, 31, 33). No heterogeneity was found. The corresponding plots are provided in Supplementary Figures 5B, 6B (7, 11, 18, 19, 23, 24, 28, 30, 31, 33).

In group C, only two clinical trials were enrolled in the final meta-analysis (29, 35). The analysis results are summarized and displayed at the bottom of Figures 5C, 6C (29, 35). No statistically significant differences were found between the studied arms. The corresponding plots are provided in Supplementary Figures 5C, 6C (29, 35).

## Discussion

With the development of antitumor immunotherapy, more and more clinical trials investigating immunotherapeutic drugs have been developed, and satisfactory clinical effects have been achieved (7–37). PD-1/PD-L1 inhibitors are currently the most widely used antitumor immunotherapeutic drugs in studied clinical trials, and they are also the most widely used antitumor immunotherapeutic drugs in clinical practice (7–37, 46, 47). In clinical trials related to PD-1/PD-L1 inhibitors, as well as in clinical work using PD-1/PD-L1 inhibitors for antitumor therapy, patients with drug-induced fever are frequently encountered and reported (7–37, 46, 47) In order to control the fever of cancer patients in a timely manner, it is important to clarify the origin of the fever (1–6). However, the specific role of PD-1/PD-L1 inhibitors in the etiology of fever is still unclear, especially when combined with chemotherapy for antitumor therapy. Therefore, when patients who are receiving combined antitumor therapy develop hyperthermia of grades 3–5, it is difficult for us to judge whether to stop the PD-1/PD-L1 inhibitor drugs to relieve the fever or not. Furthermore, due to the particularity of cancer patients, the sudden stoppage of antitumor therapy is very likely to lead to the rapid progression of the tumor. Therefore, the decision to stop or withdraw the offending drug (antitumor drug) needs to be carefully studied. In an attempt to explore the relationship between PD-1/PD-L1 inhibitors and drug-induced fever, this meta-analysis was designed and performed.

According to the guidelines of PRISMA, after screening and eligibility assessment,27 clinical trials reporting on the use of PD-1/PD-L1 inhibitors, including 15,867 cancer patients, were enrolled for the final comprehensive analysis (7–37). The quality of all included studies was evaluated and summarized in a single graph (Figure 2, Supplementary Figure 2) and was considered to be better. Therefore, the analysis conclusions drawn from the data of these clinical trials are much more inclined to be true and reliable (7–37). The data relating to fever were mainly displayed in two forms: pyrexia and febrile neutropenia. When the extracted data were verified, it was found that the incidence of pyrexia reported in clinical trials was mostly grades 1–2, while the incidence of febrile neutropenia was almost all grades 3–5 (7–37). Therefore, febrile neutropenia is the type of fever that is most likely to lead to the stoppage of the offending antitumor agent.

Comparing chemotherapy (control group), there was no statistically significant difference in the risk of pyrexia whether the PD-1/PD-L1 inhibitor was used alone or in combination with chemotherapy in the experimental arm (Figures 3A,B, 4A,B) (8–10, 12, 14, 17–24, 26–33, 35). However, we noted a statistically significant difference in the subgroup (PD-1 vs. docetaxel) (OR = 0.56, 95% CI: 0.37, 0.85; Figure 3B) (28, 30, 31). In other words, the risk of pyrexia for all-grade fever was obviously lower in the PD-1 inhibitor group compared to that of the control group among NSCLC patients (28, 30, 31). The results of the subgroup analysis suggested that the high heterogeneity (*I*^2^ = 70%; Figure 3A) might mainly originate from the two included clinical trials on NSCLC (*I*^2^ = 48%; Figure 3A) (17, 20). No publication bias was found in the funnel plot (Supplementary Figure 3A) (14, 17, 20). Through different subgrouping methods (Figure 3B, Supplementary Figure 1) (10, 12, 18, 19, 23, 24, 26–28, 30, 31, 33), we inferred that the moderate heterogeneity (*I*^2^ = 42%; Figure 3B) of the analysis results (group B) might come from all studied data themselves. The funnel plot was displayed in Supplementary Figure 3B without any clue of publication bias (10, 12, 18, 19, 23, 24, 26–28, 30, 31, 33).

Similar to the above results (Figures 3A,B), regardless of whether the control group was placebo or targeted therapy and regardless of whether PD-1/PD-L1 was used alone or in combination with targeted therapy (Figures 3C,D) (21, 22, 29, 32, 35), no statistically significant differences were found regarding the risk of pyrexia. In other words, PD-1/PD-L1 inhibitors did not result in a statistically significant increase in the risk of pyrexia. The results of the subgroup analysis suggested that the moderate heterogeneity of that analysis (*I*^2^ = 49%; Figure 3C) might originate from the included PD-1 clinical trial on gastric or junction cancer (Figure 3C) (22). Due to the small number of included clinical trials (reporting pyrexia in group D) to perform sufficient subgroup analysis, it was impossible for us to clarify the origin of low heterogeneity (*I*^2^ = 29%; Figure 3D) (8, 9).

Different from the above groups, the risk of pyrexia for the experimental group (PD-1) was higher than that of the control group (PD-1 + CTLA-4) with a statistically significant difference (OR = 2.48, 95% CI: 1.17, 5.23; Figure 3E) (29, 35). However, since only two clinical trials were enrolled in this group, the conclusion still needs to be verified by more clinical trials (29, 35). Moderate heterogeneity (*I*^2^ = 48%) was found among the included trials. The number of included trials in the above analysis was very small, and thus, it was impossible to conduct subgroup analysis to identify the origin of the resulting heterogeneity (29, 35). The corresponding funnel plot was provided in Supplementary Figure 3E (29, 35). No obvious publication bias was found.

When the same grouping and analysis methods were used to analyze the risk of pyrexia for grade 3–5 fever, no statistically significant differences were found in each group, either in the overall results or in various subgroups (Figure 4) (8, 9, 13, 14, 18, 20–24, 26–29, 31, 32, 35). In other words, PD-1/PD-L1 inhibitors did not have a statistically significant effect on the risk of pyrexia for grades 3–5 (8, 9, 13, 14, 18, 20–24, 26–29, 31, 32, 35).

Unsimilar to the trend of pyrexia risk, the majority of febrile neutropenia incidents were of grades 3–5 (7, 11, 13, 15, 16, 18, 19, 23–25, 28–31, 33, 35). Compared to chemotherapy, the risk of febrile neutropenia for all-grade fever was significantly lower in the PD-1/PD-L1 inhibitor group (OR = 0.02, 95% CI: 0.01, 0.05; Figure 5B) (7, 11, 18, 19, 23, 24, 28, 30, 31, 33). A similar trend could also be found for grades 3–5 (OR = 0.02, 95% CI: 0.01, 0.05; Figure 6B) (7, 11, 18, 19, 23, 24, 28, 30, 31, 33). On the other hand, statistically significant differences were found in other groups (Figures 5A,C, 6A,C) (13, 15, 16, 25, 29, 35). Moderate heterogeneity (*I*^2^ = 47%) was found in Figures 5A, 6A. The results of the subgroup analysis suggested that it might be related to the two enrolled clinical trials of SCLC (13, 15).

The good safety and satisfactory clinical efficacy of PD-1/PD-L1 inhibitors have been reported by a large number of clinical trials (7–37). The safety of PD-1/PD-L1 inhibitors was further confirmed by the findings of our meta-analysis. The increased risk of pyrexia for all grades could only be found when PD-1/PD-L1 plus CTLA-4 was compared with CTLA-4 alone (29, 35). Furthermore, no statistically significant differences in the risk of febrile neutropenia could be found in all studied groups, except for the PD-1/PD-L1 group, which was associated with a significantly lower risk of febrile neutropenia when compared with chemotherapy (7, 11, 13, 15, 16, 18, 19, 23–25, 28–31, 33, 35). It would be helpful for us to clarify the source of antitumor drug-induced fever and adopt the best treatment regimens for cancer patients.

## Conclusions

The increased incidence risk of pyrexia for all grades could only be found when PD-1/PD-L1 plus CTLA-4 was compared with CTLA-4. Meanwhile, compared to chemotherapy, PD-1/PD-L1 inhibitors reduced the risk of febrile neutropenia.

## Data Availability Statement

The raw data supporting the conclusions of this article will be made available by the authors, without undue reservation.

## Author Contributions

YT had the right to deal with all the data and was responsible for the decision to submit the manuscript for publication. HL, DX, WW, FS, and XY had the data of all included clinical trials. DX, HL, and WW were responsible for checking and evaluating the quality of the collected data. All authors contributed to the article and approved the submitted version.

## Conflict of Interest

The authors declare that the research was conducted in the absence of any commercial or financial relationships that could be construed as a potential conflict of interest.

## Abbreviations

PRISMA: Preferred Reporting Items for Systematic Reviews and Meta-Analyses
PD-1: programmed cell death-1
PD-L1: programmed cell death ligand 1
CTLA-4: cytotoxic T-lymphocyte antigen 4
HR: hazard ratios
OR: odds ratio
RD: risk difference
CI: confidence interval
RE: random effect
NSCLC: non-small-cell lung cancer
SCLC: small cell lung cancer
OSCC: esophageal squamous cell carcinoma
HNSCC: head and neck squamous cell carcinoma
UC: urothelial cancer
BC: breast cancer
RCC: renal cell carcinoma.
