# Supplementary figures and images for: Systematic Assessment of Risk of Fever in Solid Tumor Patients Treated With PD-1/PD-L1 Inhibitors: A Systematic Review and Meta-Analysis
Source: Front Oncol. 2020 Oct 29;10:570080. doi: 10.3389/fonc.2020.570080 (PMC7658543; doi:10.3389/fonc.2020.570080)

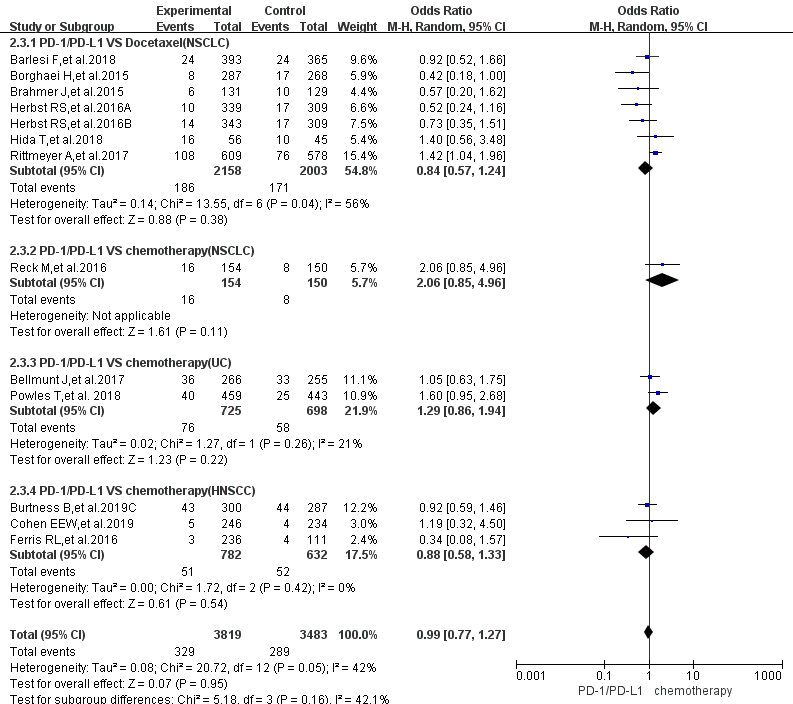

Supplement: Supplementary file 1 [file Data_Sheet_1.zip › Supplementary Figure 1.tif]

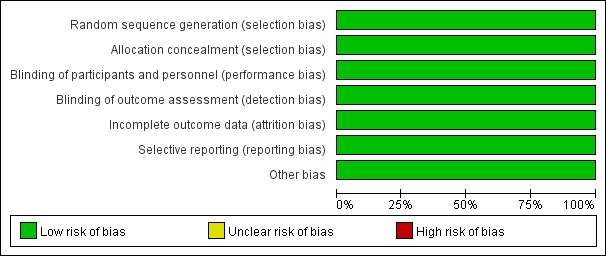

Supplement: Supplementary file 1 [file Data_Sheet_1.zip › Supplementary Figure 2.tif]

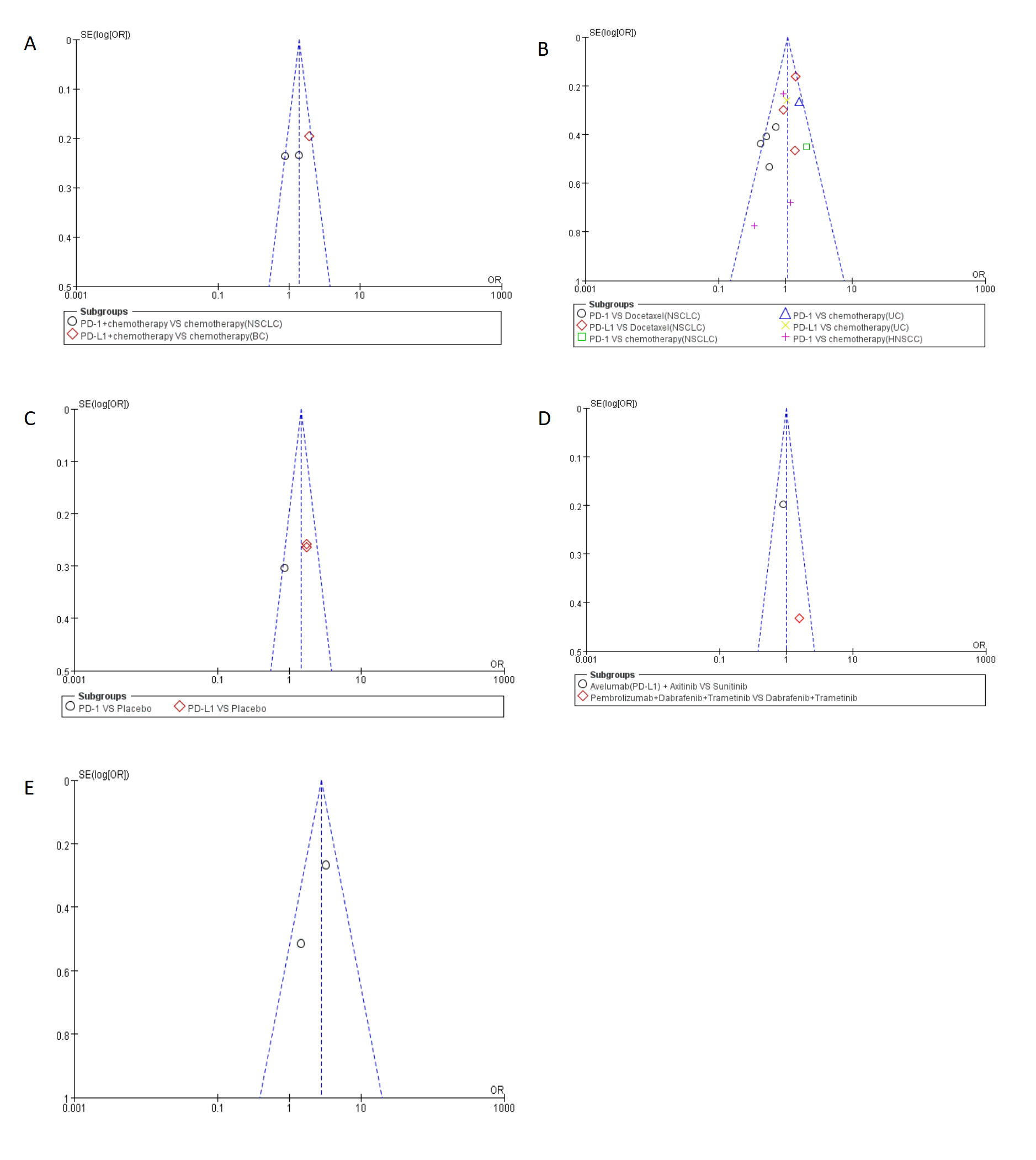

Supplement: Supplementary file 1 [file Data_Sheet_1.zip › Supplementary Figure 3.tif]

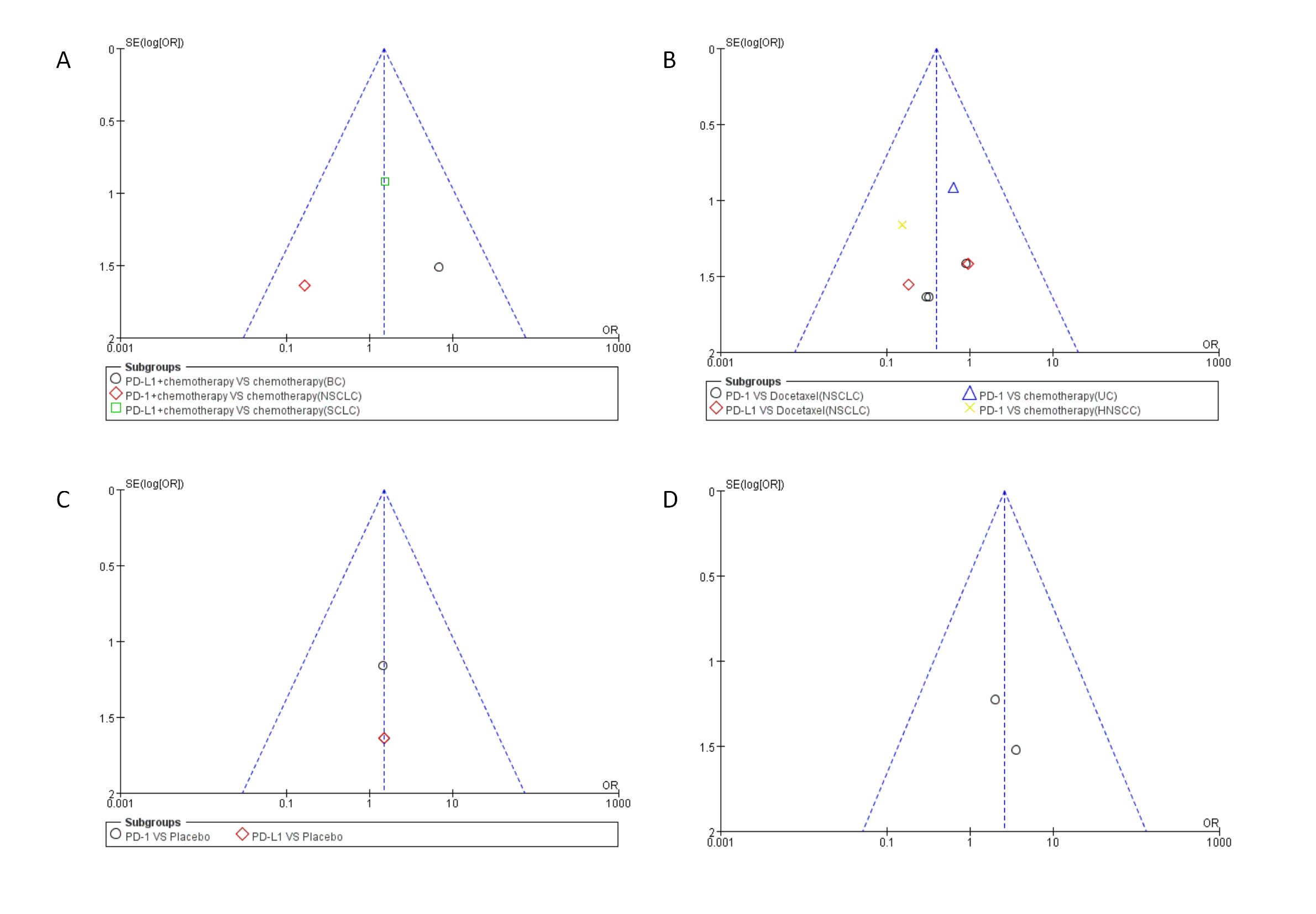

Supplement: Supplementary file 1 [file Data_Sheet_1.zip › Supplementary Figure 4.tif]

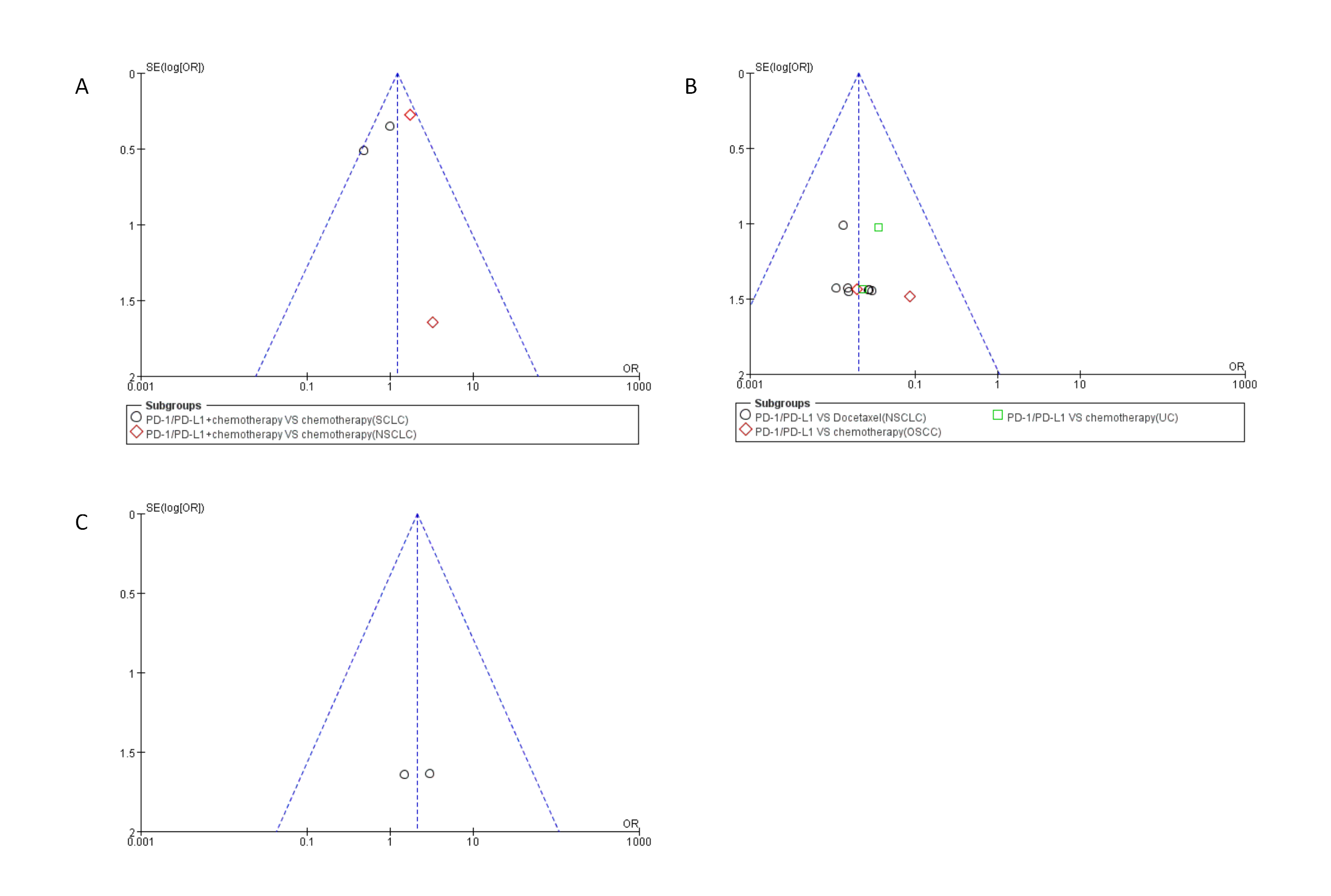

Supplement: Supplementary file 1 [file Data_Sheet_1.zip › Supplementary Figure 5.tif]

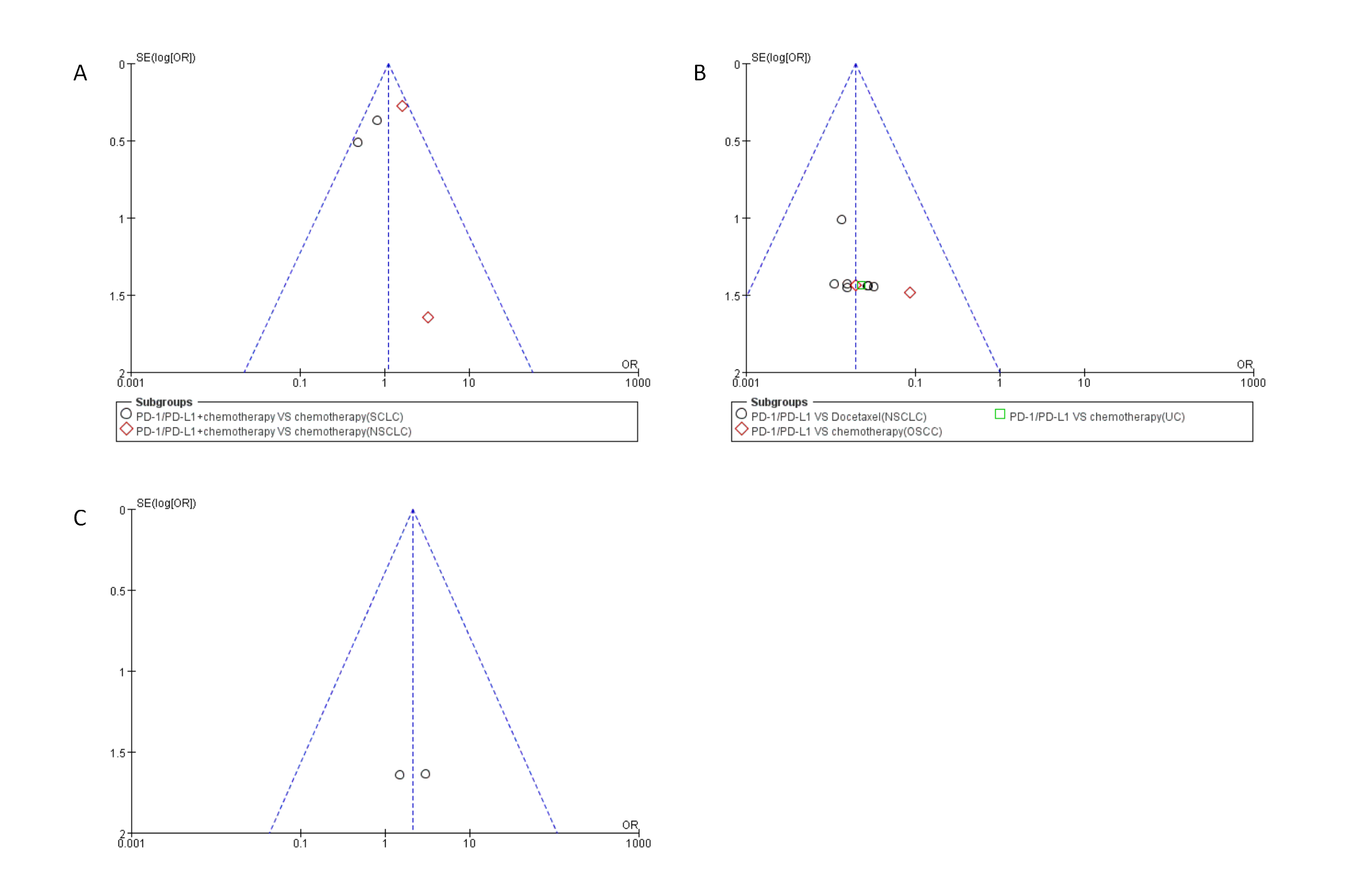

Supplement: Supplementary file 1 [file Data_Sheet_1.zip › Supplementary Figure 6.tif]
